# Comparison between clinical significance of serum CXCL-8 and classical tumor markers in oesophageal cancer (OC) patients

**DOI:** 10.1007/s10238-019-00548-9

**Published:** 2019-02-28

**Authors:** Marta Łukaszewicz-Zając, Sara Pączek, Paweł Muszyński, Mirosław Kozłowski, Barbara Mroczko

**Affiliations:** 1 Department of Biochemical Diagnostics, Medical University of Białystok, ul. Waszyngtona 15 a, 15-269 Białystok, Poland; 2 Department of Neurodegeneration Diagnostics, Medical University of Białystok, Białystok, Poland; 3 Department of Thoracic Surgery, Medical University of Białystok, Białystok, Poland

**Keywords:** Chemokines, CXCL8, Oesophageal, Tumor

## Abstract

C-X-C motif chemokine 8 (CXCL-8), known as interleukin-8, is a pro-inflammatory cytokine which acts as a chemotactic factor, mainly for leukocytes. CXCL-8 is produced by malignant cells, and therefore it can stimulate the growth and progression of various neoplasms, including oesophageal cancer (OC). The aim of the current study was to measure serum concentrations of chemokine CXCL-8 in OC patients and establish whether this protein might be considered a potential candidate for a tumor marker in the diagnosis and progression of OC. The study included 50 OC subjects (32 patients with squamous cell carcinoma of oesophagus—OSCC, 18 patients with adenocarcinoma—OAC) and 26 healthy volunteers. Serum CXCL-8 concentrations were measured using immunoenzymatic assay (ELISA). CRP levels were determined by immunoturbidimetric method, while classical tumor marker levels were measured using chemiluminescent immunoassay. CXCL-8 concentrations were significantly higher in OC patients compared to healthy controls. We demonstrated significant differences between CXCL-8 concentrations and depth of tumor invasion (T factor) in OC patients and OSCC subgroup. In addition, CXCL-8 levels were found to correlate positively with T factor and CRP concentrations. The diagnostic sensitivity, negative predictive value and the area under ROC curve (AUC) of CXCL-8 were higher than those of classical tumor markers. Our findings suggest the potential usefulness of CXCL-8 in the diagnosis and progression of OC. However, due to the non-specific nature of this chemokine, further research is needed to clarify the usefulness of CXCL-8 as a tumor marker of OC.

## Introduction

Chemokines are small proteins which play an important role in the growth, differentiation and activation of cells involved in immune response. Structurally, these proteins are classified into four subgroups, based on the arrangement of N-terminal cysteine residues (CXC, CX3C, CC, and C). A growing body of evidence suggests that these proteins may facilitate the progression of a number of malignancies. Chemokines play a role in communication between malignant cells and non-neoplastic cells within the tumor microenvironment and may promote the infiltration and activation of neutrophils and tumor-associated macrophages (TAMs) [1–7].

C-X-C motif chemokine 8 (CXCL-8), known as interleukin 8 (IL-8), belongs to a subfamily of CXC chemokines in which two N-terminal cysteines are separated by one amino acid (X) [5, 7]. This protein binds to the cell surface of G protein-coupled receptors (CXCR-1 and CXCR-2) and activates multiple intracellular signaling pathways [8, 9]. CXCL-8 has been classified as neutrophil chemoattractant, but it may also play a role in tumor progression via the regulation of angiogenesis, growth, proliferation and survival of malignant cells, including OC [5–10]. Increased CXCL-8 expression has been found in endothelial cells, infiltrating neutrophils, tumor-associated macrophages and cancer cells, including oesophageal cancer cells [8]. Some authors using immunohistochemical techniques have demonstrated that the overexpression of CXCL-8 and its specific receptor CXCR-2 is associated with tumor progression and metastasis and therefore may be a useful indicator for the survival of patients with oesophageal squamous cell carcinoma (OSCC) [11].

Oesophageal cancer (OC) is one of most common malignancy in the world. Mortality rates of this tumor are similar to incidence rates, due to its rapid progression and late-stage diagnosis [12]. Therefore, despite advances in diagnosis and treatment, the prognosis for OC patients remains poor [13, 14]. The standard methods of OC detection are endoscopic ultrasonography or computed tomography, although they have limited ability to detect microscopic lymph node metastases. Therefore, routine clinical practice requires low-cost methods of OC diagnosis [8, 13]. Several biochemical markers such as squamous cell cancer antigen (SCC-Ag) and carcinoembryonic antigen (CEA) have been investigated in OC diagnostics and follow-up of OC patients. Increased levels of the classical tumor markers predict OC recurrence and progression, but their sensitivity and specificity is not satisfactory [8–14]. Therefore, other biochemical indicators are necessary in the diagnosis of OC. According to our knowledge, the present study is the first to indicate the diagnostic usefulness of CXCL-8 levels in the sera of patients with OC in comparison with the classical tumor markers (CEA and SCC-Ag) and the well-established marker of inflammation—C-reactive protein (CRP). Therefore, the aim of the current study was to measure serum concentrations of chemokine CXCL-8 in OC patients and establish whether this protein might be considered a potential candidate for a tumor marker in the diagnosis and progression in OC patients. We expect that serum CXCL-8 levels will be significantly different in OC patients in comparison with healthy volunteers and the measurement of these chemokine concentrations might be useful in the diagnosis and progression of OC. This study is a continuation of our previous research in which we assessed the diagnostic and prognostic significance of the CXCL-8 receptor (CXCR-2) in OC patients [15].

## Methods and materials

The study group included 50 patients with OC (32 patients with OSCC and 18 patients with OAC, aged 36–82 years). The disease was diagnosed in all the studied patients in the Department of Thoracic Surgery of Bialystok University Hospital, Poland. The diagnosis of OC was based on the microscopic examination of tissue samples. OC patients were staged according to the TNM classification (tumor–nodules–metastasis), proposed by the International Union Against Cancer (UICC) [16]. Moreover, they were grouped according to tumor stage (TNM), depth of tumor invasion (T factor), the presence of lymph node (N factor) and distant metastases (M factor) as well as the histological grade (G factor) of the tumor. The characteristics of OC patients are presented in Table 1. All patients gave informed consent, and the present project was approved by the Local Ethics Committee (R-I-002/42/2015) of the Medical University of Bialystok (Poland). The control group included 26 healthy volunteers (13 females and 13 males, aged 22–66 years).

**Table 1:** Characteristics of oesophageal cancer patients

Variable tested	Number of patients
Group	
Oesophageal cancer	50
Gender	
Male	46
Female	4
Type of cancer	
Adenocarcinoma	18
Squamous cell carcinoma	32
TNM stage	
I + II	11
III	20
IV	19
Depth of tumor invasion (T factor)	
T1 + T2	10
T3	29
T4	11
Nodal involvement (N factor)	
N0	13
N1	37
Distant metastases (M factor)	
M0	38
M1	12

Blood samples of study group patients were obtained prior to treatment between 2006 and 2010 and stored at − 80 °C until assayed. CXCL-8 concentrations were measured in the patients’ sera using ELISA (enzyme-linked immunosorbent assay) kits (R&D Systems, Abingdon, UK) according to the manufacturer’s instructions. Serum levels of the classical tumor markers (CEA and SCC-Ag) were measured by the chemiluminescent microparticle immunoassay (CMIA) method using ARCHITECT 8200 ci (Abbott, USA) and for the analysis of CRP concentrations, the immunoturbidimetric method was used.

### Statistical analysis

The concentrations of the tested proteins did not follow a normal distribution in the preliminary statistical analysis (*χ*^2^-test) and nonparametric statistical analyses were performed. The Mann–Whitney test was used for the analysis of two groups, whereas the Kruskal–Wallis test was performed to compare three or more groups. In addition, the post hoc Dwass–Steel–Critchlow–Fligner was employed in the assessment of significant differences. The differences were considered to be statically significant when *p* < 0.05. Correlations were determined using the Spearman method. In addition, diagnostic characteristics such as diagnostic sensitivity, specificity, accuracy, predictive value for positive (PPV) and negative (NPV) results as well as the area under the ROC curve (AUC) of a protein tested were calculated. Statistical analysis was performed using the STATISTICA 5.1 PL program (StatSoft Inc., Tulsa, OK, USA) and for diagnostic characteristics—MedCalc statistical software (Acacialaan, Ostend, Belgium) and Microsoft Office Excel were used. Reference cutoff values were estimated using Youden Index as 12.89 pg/ml for CXCL-8; 2.85 mg/l for CRP, 4.57 ng/ml for CEA and 1.70 ng/ml for SCC-Ag).

## Results

**Fig. 1:**
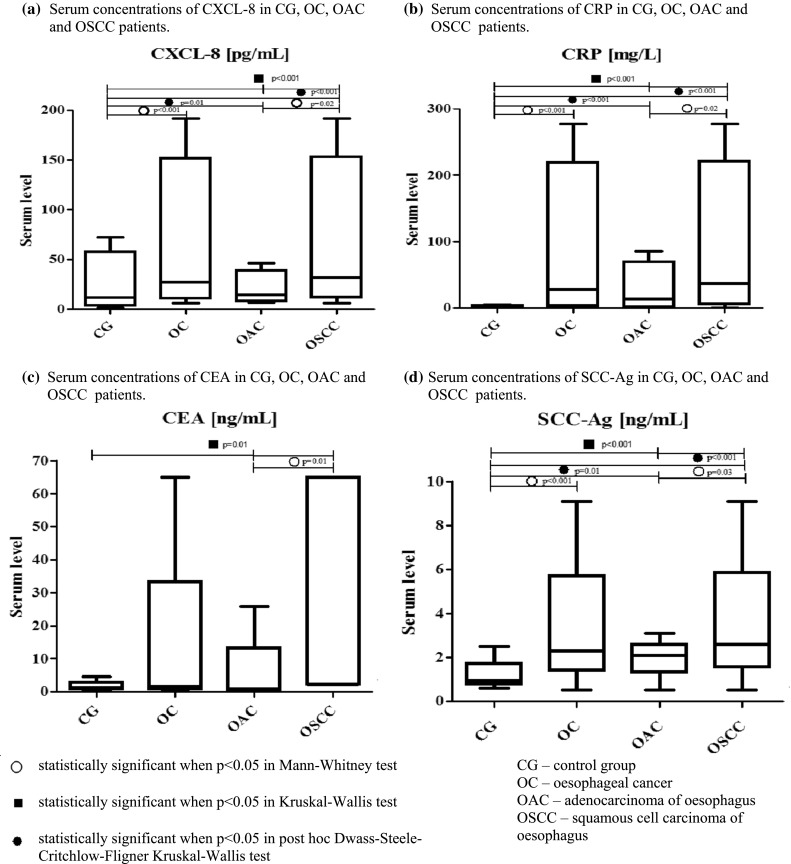
Serum levels of proteins tested in patients with oesophageal cancer (OC) and in relation to its histological types (oesophageal squamous cell cancer—OSCC and oesophageal adenocarcinoma—OAC patients) in comparison with healthy controls

Serum concentrations of chemokine CXCL-8 as well as the classical tumor markers (CEA and SCC-Ag) and the marker of inflammatory states—CRP in OC patients and healthy volunteers (control group), are presented in Fig. 1. Serum CXCL-8 levels were significantly higher (*p* < 0.001) in OC patients when compared to the healthy controls. Concentrations of the classical tumor markers (CEA and SCC-ag) as well as CRP were also found to be higher in OC patients in comparison with the control group, although only CRP (*p* < 0.001) and SCC-Ag (*p* < 0.001) levels were demonstrated to be of statistical significance (Fig. 1).

If we consider the relationship between serum concentrations of the tested proteins and the histological type of OC, CXCL-8 levels, similarly to those of the classical tumor markers and CRP, were significantly higher in patients with OSCC in comparison with subjects with OAC (Fig. 1). In addition, significant differences in CXCL-8, CRP and SCC-Ag concentrations were demonstrated between the OSCC subgroup and healthy controls, similarly to patients with OAC (Fig. 1).

**Fig. 2:**
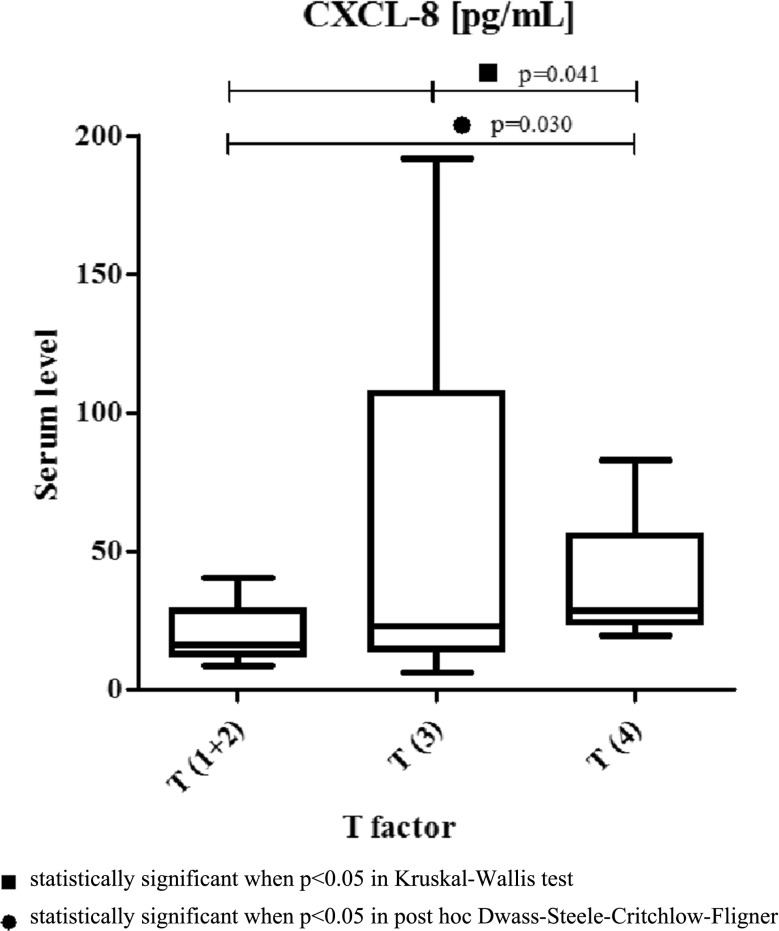
Serum concentrations of chemokine CXCL8 in patients with oesophageal cancer in relation to depth of tumor invasion (T factor)

The assessment of the relationship between the tested protein concentrations, the clinicopathological parameters and TNM stage of OC revealed that serum CXCL-8 concentrations increased with tumor stage, although these differences were not statistically significant (Table 2). Among the classical tumor markers and CRP, the differences between TNM stages were statistically significant only for SCC-Ag levels (Table 2). In addition, the differences between serum CXCL-8 levels and the depth of tumor invasion (T factor) were significant (*p* = 0.041). Additionally, the concentrations of chemokine CXCL-8 were significantly higher in T4 patients compared to the T1 + T2 subgroup (*p* = 0.030) (Table 2, Fig. 2). Moreover, statistically significant differences were found also between the depth of tumor invasion (T factor) and CRP (*p* = 0.021) as well as between T factor and SCC-Ag levels (*p* = 0.009) (Table 2).

**Table 2:** Serum concentrations of proteins tested in relation to clinicopathological parameters of oesophageal cancer (OC)

Group tested	CXCL-8 (pg/ml)	CRP (mg/l)	CEA (ng/ml)	SCC-Ag (ng/ml)
TNM stage (1 + 2)				
Median	16.65	8.50	1.50	2.20
Range	8.55–120.66	0.50–109.10	0.20–65.06	0.50–6.70
TNM (3)				
Median	19.72	5.00	1.51	2.00
Range	6.34–191.80	0.20–85.80	0.20–25.80	0.50–3.10
TNM (4)				
Median	28.60	23.60	1.60	2.90
Range	11.10–82.82	2.70–277.00	0.10–6.30	0.70–9.10
p^b^ Kruskal–Wallis test	0.16	0.12	0.76	0.01
P^c^ post hoc Dwass–Steel–Critchlow–Fligner Kruskal–Wallis test				
1 + 2 versus 3	0.85	0.98	0.92	0.504
1 + 2 versus 4	0.24	0.32	0.99	0.32
3 versus 4	0.27	0.12	0.73	0.004
T factor (T1 + 2)				
Median	16.28	6.25	1.49	2.50
Range	8.55–40.72	0.50–109.10	0.20–10.10	0.50–6.70
T factor (T3)				
Median	22.76	6.00	1.40	2.10
Range	6.34–191.80	0.20–85.80	0.20–65.06	0.50–5.00
T factor (T4)				
Median	28.60	35.90	2.10	2.90
Range	19.58–82.82	5.20–277.00	0.10–5.00	0.70–9.10
p^b^ Kruskal–Wallis test	0.041	0.021	0.77	0.009
p^c^ post hoc Dwass–Steel–Critchlow–Fligner Kruskal–Wallis test				
1 + 2 versus 3	0.367	0.939	0.901	0.196
1 + 2 versus 4	0.030	0.037	0.696	0.630
3 versus 4	0.224	0.033	0.930	0.010
N factor (N0)				
Median	16.65	8.50	1.47	2.20
Range	8.55–120.66	0.50–126.30	0.20–65.06	0.50–9.10
N factor (N1)				
Median	25.07	11.00	1.62	2.50
Range	6.34–191.80	0.20–277.00	0.10–25.80	0.50–5.80
p^a^ Manna–Whitney test	0.36	0.60	0.86	0.90
M factor (M0)				
Median	20.35	8.30	1.66	2.20
Range	6.34–191.80	0.20–126.30	0.10–65.06	0.50–9.10
M factor (M1)				
Median	28.14	13.05	1.28	2.55
Range	11.10–62.46	2.70–277.00	0.63–6.30	0.70–5.00
p^a^ Manna–Whitney test	0.34	0.34	0.95	0.42

^a^Statistically significant when *p* < 0.05 in Mann–Whitney test

^b^Statistically significant when *p* < 0.05 in Kruskal–Wallis test

^c^Statistically significant when *p* < 0.05 in post hoc Dwass–Steel–Critchlow–Fligner Kruskal–Wallis test

Spearman’s correlation indicated that there was a significant correlation between serum CXCL-8 concentrations, depth of tumor invasion (T factor) (*p* = 0.010) and CRP concentrations (*p* = 0.003), while a positive correlation existed between SCC-Ag concentrations and TNM stage (*p* = 0.030) (Table 3). When we considered correlations between the clinicopathological features of the tumor and serum levels of the tested proteins in relation to the histological type of OC, we found a positive correlation only between serum CXCL-8 levels and CRP concentrations (*p* = 0.021) (data not shown).

**Table 3:** Correlations between clinicopathological features of tumor and serum levels of proteins testes in patients with oesophageal cancer

	Age	TNM	T	G	Tumor length	CXCL-8	CRP	CEA	SCC-Ag
Age									
*r*	1.00	− 0.03	0.06	0.06	− 0.09	0.07	− 0.10	− 0.17	− 0.24
*p*		> 0.05	> 0.05	> 0.05	> 0.05	> 0.05	> 0.05	> 0.05	> 0.05
TNM									
*r*	− 0.03	1.00	0.80	0.27	0.67	0.27	0.25	0.05	0.31
*p*	> 0.5		< 0.001	> 0.05	< 0.001	> 0.05	> 0.05	> 0.05	0.03
T									
*r*	0.06	0.80	1.00	0.29	0.61	0.36	0.35	0.10	0.15
*p*	> 0.05	< 0.001		0.04	< 0.001	0.01	0.01	> 0.05	> 0.05
G									
*r*	0.06	0.27	0.29	1.00	0.35	0.22	0.24	− 0.11	0.14
*p*	> 0.05	> 0.05	0.04		0.01	> 0.05	> 0.05	> 0.05	> 0.05
Tumor length									
*r*	− 0.09	0.67	0.61	0.35	1.00	0.18	0.22	0.06	0.04
*p*	> 0.05	< 0.001	< 0.001	0.01		> 0.05	> 0.05	> 0.05	> 0.05
CXCL-8									
*r*	0.07	0.27	0.36	0.22	0.18	1.00	0.41	0.11	0.15
*p*	> 0.05	> 0.05	0.01	> 0.05	> 0.05		0.01	> 0.05	> 0.05
CRP									
*r*	− 0.10	0.25	0.35	0.24	0.22	0.41	1.00	0.17	0.32
*p*	> 0.05	> 0.05	0.01	> 0.05	> 0.05	0.01		> 0.05	0.03
CEA									
*r*	− 0.17	0.05	0.10	− 0.11	0.06	0.11	0.17	1.00	0.21
*p*	> 0.05	> 0.05	> 0.05	> 0.05	> 0.05	> 0.05	> 0.05		> 0.05
SCC-Ag									
*r*	− 0.24	0.31	0.15	0.14	0.04	0.15	0.32	0.21	1.00
*p*	> 0.05	0.03	> 0.05	> 0.05	> 0.05	> 0.05	0.03	> 0.05	

The percentage of elevated concentrations (diagnostic sensitivity) of CXCL-8 (86%) was higher than that of CRP (82%) and far higher than those of the classical tumor markers: SCC-Ag (74%) and CEA (18%) (Table 4). The highest diagnostic sensitivity was obtained for the combined measurement of CXCL-8 and CRP (100%), and this value was higher than that for the combined measurement of the classical tumor markers (76%) (Table 4). The diagnostic specificity of CXCL-8 levels (73%) was lower than that of the classical tumor markers and CRP. The positive predictive value (PPV) was lowest for CXCL-8 levels (86%) among all the tested proteins, but the negative predictive value (NPV) for CXCL-8 (73%) was the same as for CRP and higher than for SCC-Ag (66%) and CEA (39%). The diagnostic accuracy of CXCL-8 (82%) was marginally lower than that of CRP (86%), but higher than that of CEA, and increased to 88% in the combined measurement with CRP or SCC-Ag (Table 4).

**Table 4:** Diagnostic criteria for CXCL-8. Classical tumor markers (CEA and SCC-Ag) and C-reactive protein (CRP) levels in oesophageal cancer (OC) patients

	Diagnostic sensitivity (%)	Diagnostic specificity (%)	PPV (%)	NPV (%)	Accuracy (%)
CXCL-8	86	73	86	73	82
CRP	82	92	95	73	86
CEA	18	100	100	39	46
SCC-Ag	74	96	97	66	82
CXCL-8 + CRP	100	65	85	100	88
CXCL-8 + CEA	86	73	86	73	82
CXCL-8 + SCC	96	73	87	90	88
CRP + CEA	84	92	95	75	87
CRP + SCC	90	88	94	82	89
CEA + SCC	76	96	97	68	83

**Fig. 3:**
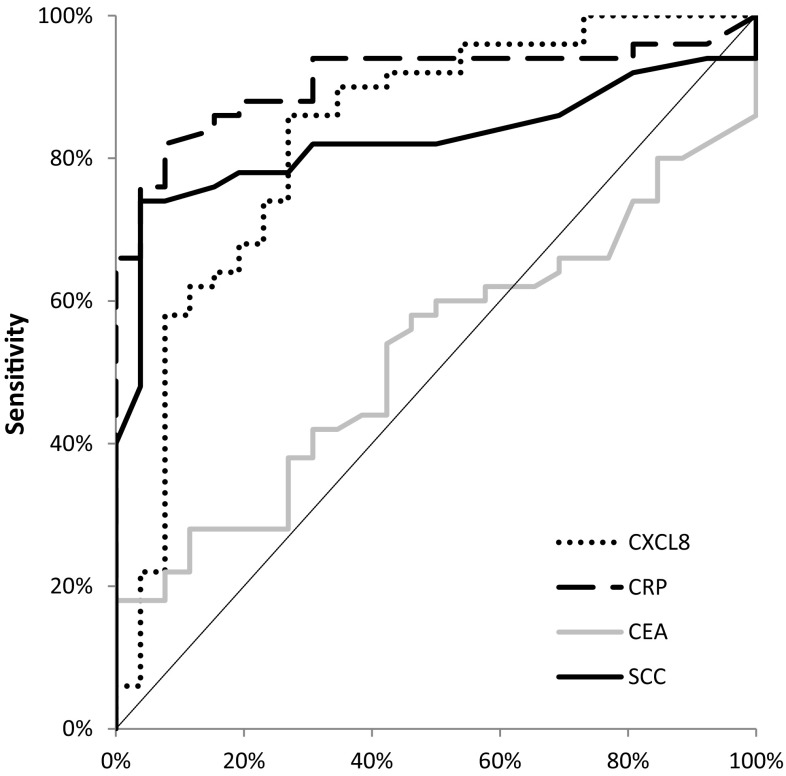
Areas under ROC curves (AUC) for chemokine CXCL-8 (0.8315, *p* < 0.001), C-reactive protein (0.9092, *p* < 0.001), carcinoembryonic antigen (0.5238, *p* > 0.05), squamous cell cancer antigen (0.8250, *p* < 0.001) in oesophageal cancer (OC) patients

**Fig. 4:**
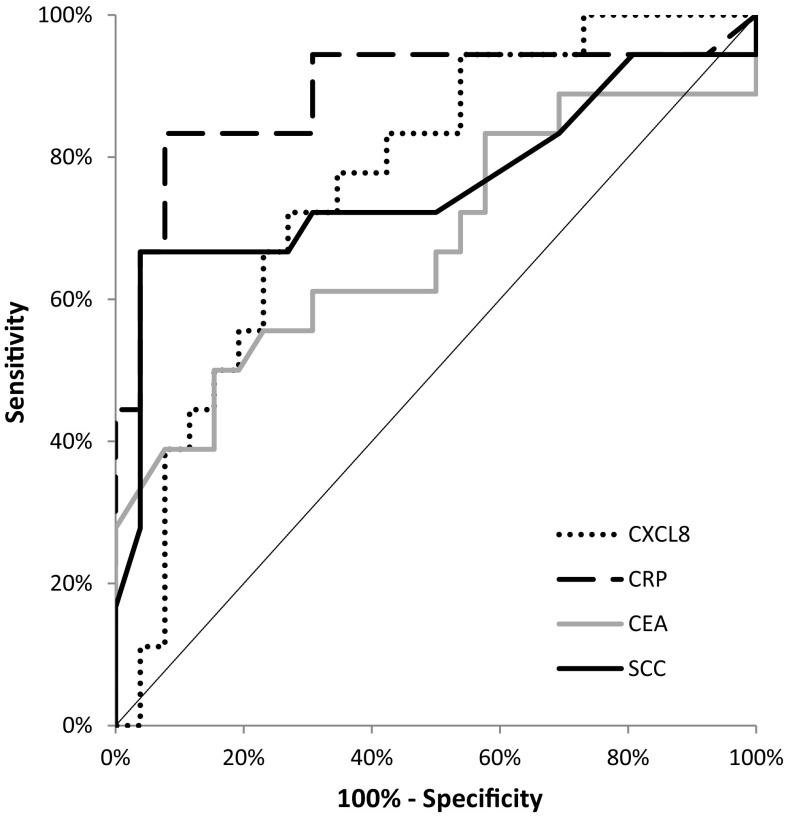
Areas under ROC curves (AUC) for chemokine CXCL-8 (0.7650, *p* < 0.001), C-reactive protein (0.8910, *p* < 0.001), carcinoembryonic antigen (0.6784, *p* = 0.046), squamous cell cancer antigen (0.7618, *p* < 0.001) in oesophageal adenocarcinoma (OAC) patients

**Fig. 5:**
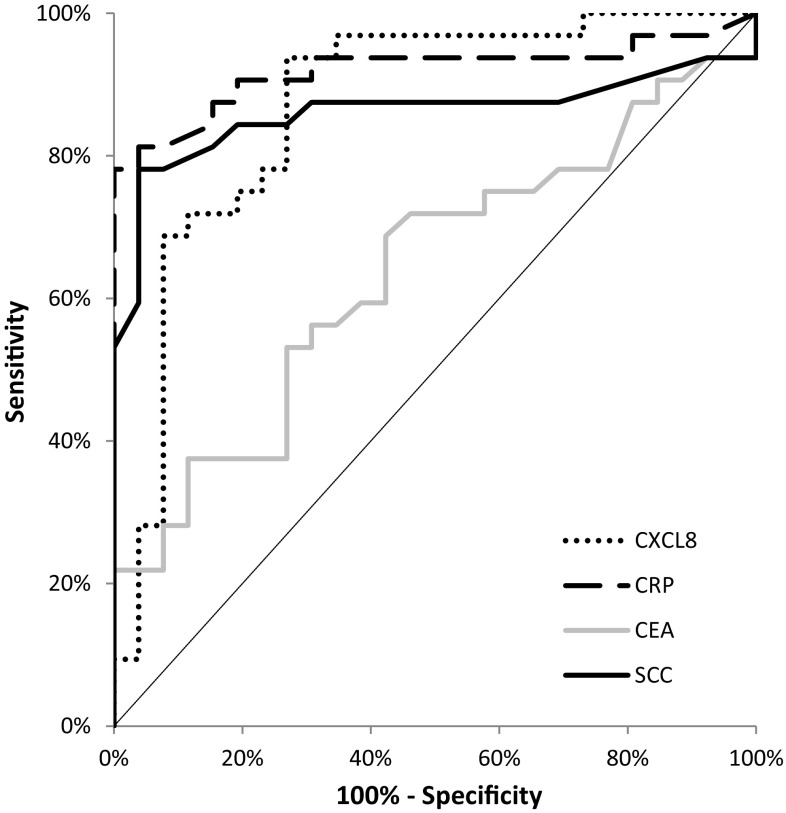
Areas under ROC curves (AUC) for chemokine CXCL-8 (0.8690, *p* < 0.001), C-reactive protein (0.9195, *p* < 0.001), carcinoembryonic antigen (0.6376, *p* = 0.06), squamous cell cancer antigen (0.861, *p* < 0.001) in oesophageal squamous cell cancer (OSCC) patients

The area under the ROC curve (AUC) indicates the clinical usefulness of the tested proteins in cancer diagnosis. In the total OC group, the AUC of CXCL-8 (0.8315; *p* < 0.001) was lower than the AUC of CRP (0.9092; *p* < 0.001), but higher in comparison with the AUCs of the classical tumor markers (CEA = 0.5238; *p* = 0.719 and SCC-Ag = 0.8250; *p* < 0.001) in the diagnosis of OC (Fig. 3). Similar results were obtained for the OSCC and OAC subgroups, where the AUC of CXCL-8 was also lower than that of CRP and higher than those of the classical tumor markers (Figs. 4, 5).

## Discussion

Several proteins have been proposed as candidates for biomarkers of OC. However, they cannot be applied in clinical practice due to their insufficient specificity and sensitivity. Non-invasive methods such as measuring serum levels of biochemical markers provide a possibility for formatting a panel of biomarkers which may play an important role in cancer management including diagnostic procedures [8–14].

Oesophageal cancer belongs to the most aggressive malignant diseases, and therefore new diagnostic tools are critically needed. A number of clinical investigations have suggested the importance of selected chemokines, such as CXCL-8, in OC progression. However, these studies have predominantly assessed expression levels of chemokines in OC tissue [5, 8, 10, 11, 13]. The present study is a continuation of our previous research in which we established the usefulness of a specific receptor for CXCL-8—CXCR-2 in the diagnosis and prognosis of OC [15]. Therefore, in our present study, we evaluated whether serum CXCL-8 levels might be used as a potential tumor marker for OC. We indicated that serum CXCL-8 levels were significantly higher in OC patients when compared to healthy controls as well as in patients with OSCC in comparison with subjects with OAC. Similar results have been published by other authors who studied OSCC patients and revealed that serum CXCL-8 concentrations were significantly higher in OSCC patients than in healthy controls [17]. Furthermore, in OSCC patients with increased serum CXCL-8 levels, CXCL-8 and its specific receptor (CXCR-2) were also overexpressed [11]. The authors concluded that there was a direct connection between CXCL-8 secreted from OSCC cells and increased serum CXCL-8 levels [11]. Moreover, elevated concentrations of CXCL-8 were also found in OAC patients when compared to healthy controls, although the study was performed using a multiplexed assay [18]. Our present data, as well as other authors’ investigations, have indicated that CXCL-8 might be synthesised by OC cells.

In our present study, we assessed the relationship between serum CXCL-8 levels and clinicopathological characteristics of the tumor. Similarly to other authors, we failed to establish any significant correlations between CXCL-8 concentrations and TNM stage [17]. However, we demonstrated significant differences between serum CXCL-8 levels and depth of tumor invasion (T factor). In our previous study concerning the measurement of concentrations of a specific receptor for CXCL-8 (CXCR-2), we found statistically significant differences only between serum CXCR-2 levels and tumor differentiation (G factor) [15]. Opposite results were obtained using the immunohistochemical method, where the authors demonstrated that the increased expression of both CXCL-8 and CXCR-2 correlated with the depth of invasion, lymph node metastasis, pathological stage, lymphatic and venous invasion [11].

In the present study, we established significant correlations between serum CXCL-8 concentrations, depth of tumor invasion (T factor) and CRP concentrations in OC patients and the OSCC subgroup. Ogura et al. [11] also revealed that serum CXCL-8 concentrations correlated with the biochemical marker of inflammation—CRP. The authors concluded that circulating CXCL-8 was associated with the inflammatory status of OSCC [11].

According to our knowledge, the present study is the first to assess the diagnostic significance of serum CXCL-8 in OC patients. In our data, the percentage of elevated concentrations (diagnostic sensitivity) and the negative predictive value (NPV) of CXCL-8 was higher than those of the classical tumor markers. The highest diagnostic sensitivity was obtained for the combined measurement of CXCL-8 and CRP. Furthermore, the positive predictive value (PPV) for CXCL-8 levels was higher than that for SCC-Ag, but lower than that for CRP and CEA, while the diagnostic accuracy of CXCL-8 was marginally lower than that of CRP, but higher than that of the classical tumor markers. Our previous study proved that the diagnostic sensitivity, accuracy and predictive value of negative results for serum CXCR-2 levels were higher than those for CEA and SCC-Ag, and marginally lower than those for CRP concentrations [15]. Similarly to the present study, the highest diagnostic sensitivity was found for the combined analysis of CXCR-2 and CRP [15]. In our present paper, we also assessed the clinical usefulness of the tested proteins in disease diagnosis by calculating the area under the ROC curve (AUC). We revealed that in the total OC group, the AUC of CXCL-8 was lower than that of CRP, but higher in comparison with the AUCs of the classical tumor markers in OC diagnosis. Similar results were obtained for the OSCC and OAC subgroups. Our previous findings indicted that the AUC of CXCR‑2 in OC patients was higher than that of SCC‑Ag and marginally lower than those of CRP and CEA [15].

## Conclusions

According to our knowledge, the present study is the first to compare the diagnostic characteristics of CXCL-8 with the well-established tumor markers (CEA and SCC-Ag) and the marker of inflammation—CRP in the sera of OC patients. The percentage of elevated concentrations and the most important diagnostic criterion, AUC of CXCL-8, was higher than the AUCs of the classical tumor markers. Moreover, in the present paper we assessed serum CXCL-8 levels in relation to the clinicopathological features of OC and proved statistically significant differences between serum CXCL-8 levels and depth of tumor invasion (T factor). In addition, the concentrations of this chemokine positively correlated with T factor as well as CRP. Our findings suggest the potential usefulness of serum CXCL-8 in the diagnosis and progression of OC. However, due to the non-specific nature of this chemokine, further research is needed to clarify the usefulness of CXCL-8 as a potential tumor marker of OC.
